# Neutral Axis Depth versus Ductility and Plastic Rotation Capacity on Bending in Lightweight-Aggregate Concrete Beams

**DOI:** 10.3390/ma12213479

**Published:** 2019-10-24

**Authors:** Luís Bernardo, Miguel Nepomuceno, Hugo Pinto

**Affiliations:** 1 C-MADE-Centre of Materials and Building Technologies, Department of Civil Engineering and Architecture, University of Beira Interior, 6201-001 Covilhã, Portugal; lfb@ubi.pt; 2 Department of Civil Engineering and Architecture, University of Beira Interior, 6201-001 Covilhã, Portugal

**Keywords:** lightweight-aggregate concrete, reinforced concrete, beams, bending, neutral axis depth, ductility, plastic rotation capacity

## Abstract

This article presents an experimental study on the evolution of the neutral axis depth at failure in the critical section with the flexural ductility and plastic rotation capacity of reinforced concrete (RC) lightweight-aggregate concrete (LWAC) beams. For this, the results of a previous experimental program involving RC LWAC beams tested in flexure until failure are used. The variable studies were the concrete compressive strength (between 22.0 and 60.4 MPa and dry density between 1651 and 1953 kg/m^3^) and the longitudinal tensile reinforcement ratio (between 0.13% and 2.69%). The flexural ductility and the plastic rotation capacity of the RC LWAC beams are characterized by a ductility index and a plastic trend parameter, respectively. The influence of the variable studies, as well as the relation of the flexural ductility and plastic rotation capacity with the values for the neutral axis depth at failure are analyzed and discussed. Some conclusions are drawn which can be useful for the design of RC LWAC beams for flexure. In particular, it is shown that the practical rule of limiting the neutral axis depth at failure to ensure ductile behavior, as used in normal-weight aggregate concrete beams, is also valid for RC LWAC beams.

## 1. Introduction

During the last few decades, lightweight-aggregate concretes (LWAC) have deeply improved due to the evolution of the chemical admixtures and minerals. Among the successive improvements of LWAC, it is worth mentioning the workability, durability and compressive strength. Such properties, in addition to the lighter weight of the material, are important for many structural applications, such as offshore and marine structures, slabs and joists in high-rise buildings, bridge decks, rehabilitation and strengthening of existing buildings, and prefabricated constructions. 

Several studies on the properties of LWAC, as well as specific recommendations for the design of LWAC members and reports of successful applications, have been published in past years. Despite this, some aspects of the structural performance of LWAC still needs further research, namely the ductile behavior of LWAC members. This is because some mechanical properties of the concrete are modified by incorporating lightweight-aggregates (LWA) instead of normal-weight aggregates (NWA). Bogas and Gomes in 2013 [1] showed that the failure mode of LWAC depends on the concrete strength level, properties of mortar, as well as type and volume content of LWA. Domagala in 2011 [2] and Cui et al. in 2012 [3] showed that the tensile to compressive strength ratio and the fracture toughness of LWAC are lower. In fact, when compared with normal-weight aggregate concrete (NWAC), LWAC is shown to be more brittle both in tension and compression (Jung et al. in 2007 [4]).

However, the relative higher brittleness of LWAC does not necessarily results in a lower deformation capacity at the ultimate stage of reinforced concrete (RC) LWAC members. This is because such members usually combine concrete, which is a relative fragile material, with hot-rolled reinforcing steel bars which show high plasticity behavior. Such combination provides ductility for RC LWAC members. This was previously confirmed with experiments on beams under flexure which were built with LWAC (Liu et al. in 2006 [5], Bernardo et al. in 2016 [6,7]). However, such studies have also shown that some important differences exist between the performance of RC LWAC and RC NWAC beams. For instance, the flexural ductility of RC LWAC beams is lower when compared with similar RC NWAC beams. 

The drawback related with the lower flexural ductility of RC LWAC beams is important because current design codes require that structural members must insure sufficient ductility under overload conditions, in addition to the required strength. In fact, ductility is an important key property to ensure the required capacity for internal forces redistribution and structural safety, namely in seismic areas.

The flexural ductility of beams can be defined as the capacity of the member to undergo high plastic deformations in the critical regions without an appreciable loss of its loading resistance. To ensure enough ductility, the beam should be designed in order to respect the detailing rules for reinforcement provided in design codes. Besides, the ductility depends directly on the plastic rotation capacity of the critical sections, which can be achieved using some basic rules, namely:

- using steel with suitable ductility properties for the reinforcing bars;
- ensuring a proper design for the critical sections such that the neutral axis depth at failure is small (usually characterized by parameter x/d, with x the neutral axis depth at failure and d the effective depth of the longitudinal tensile reinforcement);
- using closed stirrups as transverse reinforcement, with small longitudinal spacing, to ensure a suitable confinement of concrete in the compression area of the cross-section;
- using additional longitudinal reinforcement in the compression area of the cross-section.

In addition, for the ultimate load, the formation of plastic hinges in the critical regions must be checked to ensure ductility. This is done by checking if the strains at failure of the materials (steel and concrete) are big enough to ensure the plastic behavior of the critical sections. 

Among the previously referred rules, one of the most practical is to limit the relative neutral axis depth (x/d) at failure in the critical sections. This rule is stated by several design codes and proved, over decades of experience, to be appropriate for RC NWAC beams. Controlling the depth of the compressed concrete in failure strongly depends on the characteristics of steel and also on the mechanical ratio of the longitudinal tensile reinforcement. For this reason, design codes usually also state upper limits for the referred mechanical ratio in order to allow the yielding of the tensile reinforcement before concrete crushing in the compression area of the cross-section. 

The previously referred rules are expected to be also valid for RC LWAC beams since they rely on fundamental mechanical principles for reinforced concrete. However, since differences were reported between RC LWAC and RC NWAC beams as far as ductility is concerned, specific studies on the ductility of RC LWAC beams are important to verify or correct such design rules mainly based on experimental results with RC NWAC beams. In past years, many of these rules have simply been extrapolated from RC NWAC to RC LWAC. For this reason, studies specially focused on the flexural behavior of RC LWAC beams continue to be reported in the literature, such as the ones from Liu et al. in 2006 [5], Jung et al. in 2007 [4] and Sin et al. in 2010 [8]. A more detailed literature review can be found in [6]. As a consequence, proposals for the extension of design codes to RC LWAC beams have been reported and the work is still ongoing.

However, studies specifically focused on the ductility of RC LWAC beams under flexure are still very scarce. In two recent studies from the authors (Bernardo et al. in 2016 [6,7]), the flexural ductility and the plastic behavior of RC LWAC beams were experimentally studied. Flexural ductility was characterized by ductility indexes, while plastic rotation capacity was characterized by a plastic trend parameter. Some important findings about how concrete compressive strength and longitudinal tensile reinforcement ratio influence ductility and plastic rotation capacity, and also on the appropriate range for the longitudinal tensile reinforcement ratio to ensure ductility and plastic behavior, were pointed out. In order to continue this important work, in this article the authors aim, in particular, to check specifically if the practical rule of limiting the neutral axis depth in failure at the critical sections, which is accepted and used nowadays for RC NWAC beams, is also valid for RC LWAC beams.

## 2. Previous Experimental Tests 

A detailed description of the experimental work performed by the authors can be found in previous articles (Bernardo et al. in 2016 [6,7]). However, a brief description of the used materials, tested RC LWAC beams and testing procedure is presented here.

**Figure 1:**
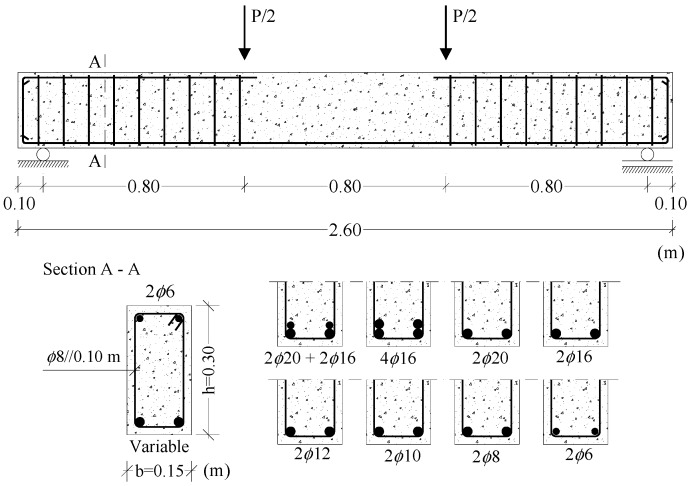
Geometry and detailing of tested reinforced concrete lightweight-aggregate concrete (RC LWAC) beams.

Nineteen RC LWAC beams were tested until failure under four-point flexural loading (Figure 1). The beams were 2.60 m long with a rectangular cross-section 0.15 m × 0.30 m. The concrete compressive strength was measured with 150 mm cube specimens and varied between 22.0 and 60.4 MPa (with dry density between 1651 and 1953 kg/m^3^), and the longitudinal tensile reinforcement ratio varied between 0.13% and 2.69%. Hot rolled ribbed steel rebars S400 were used (Figure 1). The concrete cover was 2 cm. In order to prevent shear failure in the region near the supports, sufficient transverse reinforcement (stirrups) was provided outside the central zone.

Table 1 summarizes the main properties of the tested beams, namely: the average LWAC compressive strength ($f_{lc}$), the dry density of LWAC ($\delta_{l}$), the area of longitudinal tensile reinforcement ($A_{s}$) and the adopted solutions for the steel bars (diameter ϕ is given in mm), the effective depth of the longitudinal tensile reinforcement measured from the top face (d) and the longitudinal tensile reinforcement ratio ($\rho =100\times A_{s}/bd$). The name of the beams has the following meaning: series number ($f_{lc}$-ρ). Three series were defined as function of the range for the concrete compressive strength.

LWAC was produced in laboratory. Portland cements type CEM I 42.5R and CEM II/B-L32.5N (specific gravity 3.14 and 3.04 and fineness 385 and 462 m^2^/kg, respectively) and two mineral additions, limestone powder and microsilica (specific gravity 2.72 and 2.17 and fineness 509 and 130 m^2^/kg, respectively), were used. Fine aggregates included natural normal-weight sand (Sand 0/5), with specific gravity of 2.61 and fineness modulus of 2.705. The coarse aggregate includes only lightweight expanded clay aggregates (Leca 4/12 with $D_{\max}$ = 12.7 mm) with specific gravity of 1.31, fineness modulus of 5.958 and water absorption of 14.1% at 24 h, 3.98% at 30 min and 3.56% at 15 min. A liquid superplasticizer was also used. Table 2 summarizes, for each series of beams, the mix proportions of the LWAC produced in the laboratory. The slump test was between 8 and 12 cm.

For the steel bars, the average yielding stress ($f_{y}$) was measured through uniaxial tensile tests and varied between 503 and 575 MPa. A constant Young’s Modulus (Es) of 200 GPa was assumed to compute the corresponding yielding strains values ($\varepsilon_{y}$) from Hooke’s law.

**Figure 2:**
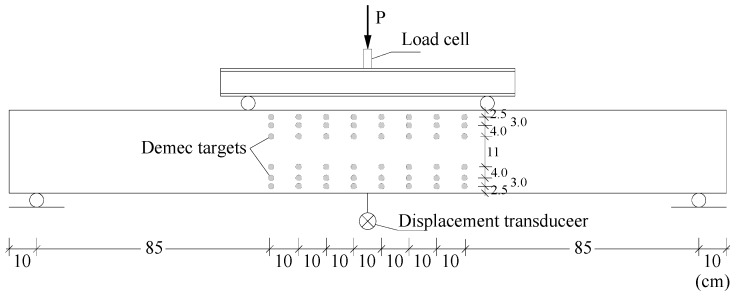
Test set-up.

Figure 2 illustrates the test set-up. To measure the horizontal strains along the height of the central cross-sections, an external grid of Demec targets were glued in the lateral face, as illustrated in Figure 2. A displacement transducer was placed at midspan to record the vertical displacement and a load cell was used to record the effective applied load. In addition, resistance strain gauges were glued in the longitudinal tensile bars (at midspan) to record the tensile strains. All tests were performed under deformation control.

**Figure 3:**
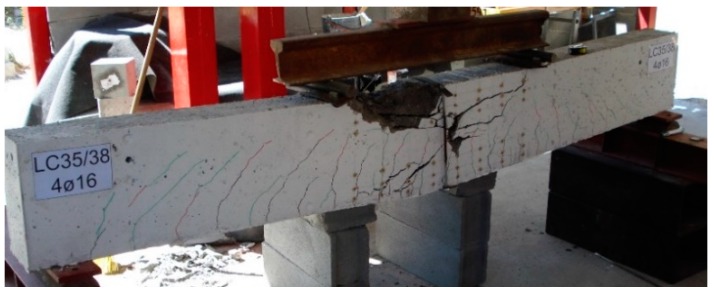
Typical failure.

As an example, Figure 3 shows a tested beam after failure. Except for the first beams of each series, the beams failed in pure flexion at the central region by crushing of concrete on the upper face. The referred first beams, with the lower reinforcement ratios, failed by the tensile longitudinal bars without the crushing of the concrete.

## 3. Previous Experimental Results

This section summarizes some of the results for the tested RC LWAC beams previously presented by the authors (Bernardo et al. [6,7]) and which are needed for the present study.

### 3.1. Load versus Deflection Curves and Deflection Ductility Indexes

**Figure 4:**
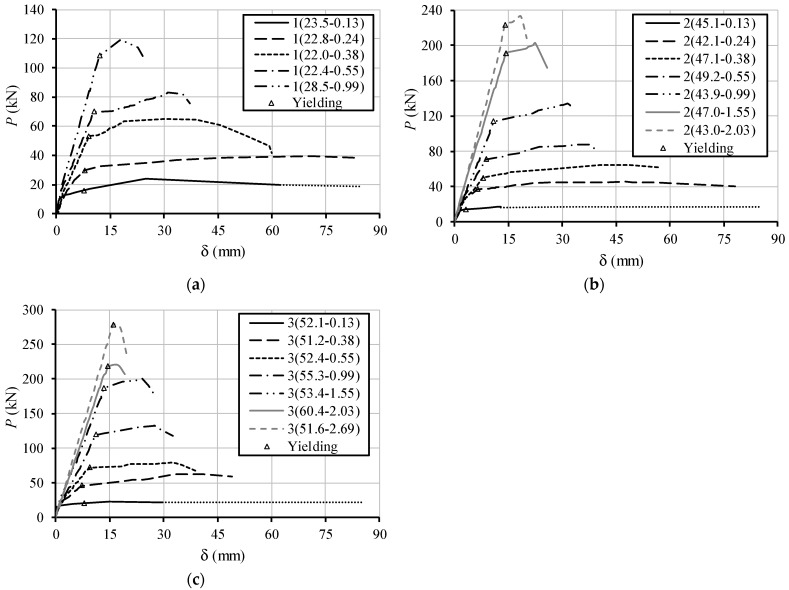
Load versus deflection curves: (**a**) series 1; (**b**) series 2; (**c**) series 3.

Figure 4 presents, for each series of tested RC LWAC beams, the total applied load (P) versus deflection at midspan (δ) curves. As previously explained by the authors [6], for the first beams of each series (with the lower reinforcement ratio), the yielding of the reinforcement suddenly occurred after cracking. For the other beams, the graphs in Figure 4 show typical shapes where the domain corresponding to each behavioral stage can be identified. Also, the observed trends are the expected ones. In particular, for the sake of this article, it can be seen that, in each series (for a given range of concrete strengths), the flexural ductility tends to decrease as the tensile reinforcement ratio increases. A detailed discussion of the behavioral curves presented in Figure 4 can be found in [6]. 

Based on the graphs presented in Figure 4, Bernardo et al. [6] characterized the flexural ductility of the beams by using a deflection ductility index. This index was defined as follows:

(1) $$\mu_{\delta }=\frac{\delta_{u}}{\delta_{y}}$$

In Equation (1), $\delta_{u}$ is the ultimate deflection corresponding to the ultimate load and $\delta_{y}$ is the yielding deflection corresponding to the yielding of the longitudinal tensile reinforcement. The ultimate deflection was defined in a conventional way from the P–δ curves (this discussion can be found in [6]), it corresponds to the deflection of the intersection point between the descending branch of the curve with a horizontal line that across the yielding point. For P–δ curves with no descending branches, the ultimate deflection is the corresponding to the last point on the curve.

Table 3 summarizes, for each tested beam, the obtained values for the deflection ductility index ($\mu_{\delta }$). The first beam of each series were not included because the last part of their experimental P–δ curves were not considered reliable to obtain $\delta_{u}$.

### 3.2. Plastic Rotation versus Deflection Curves and Plastic Trend Parameters

**Figure 5:**
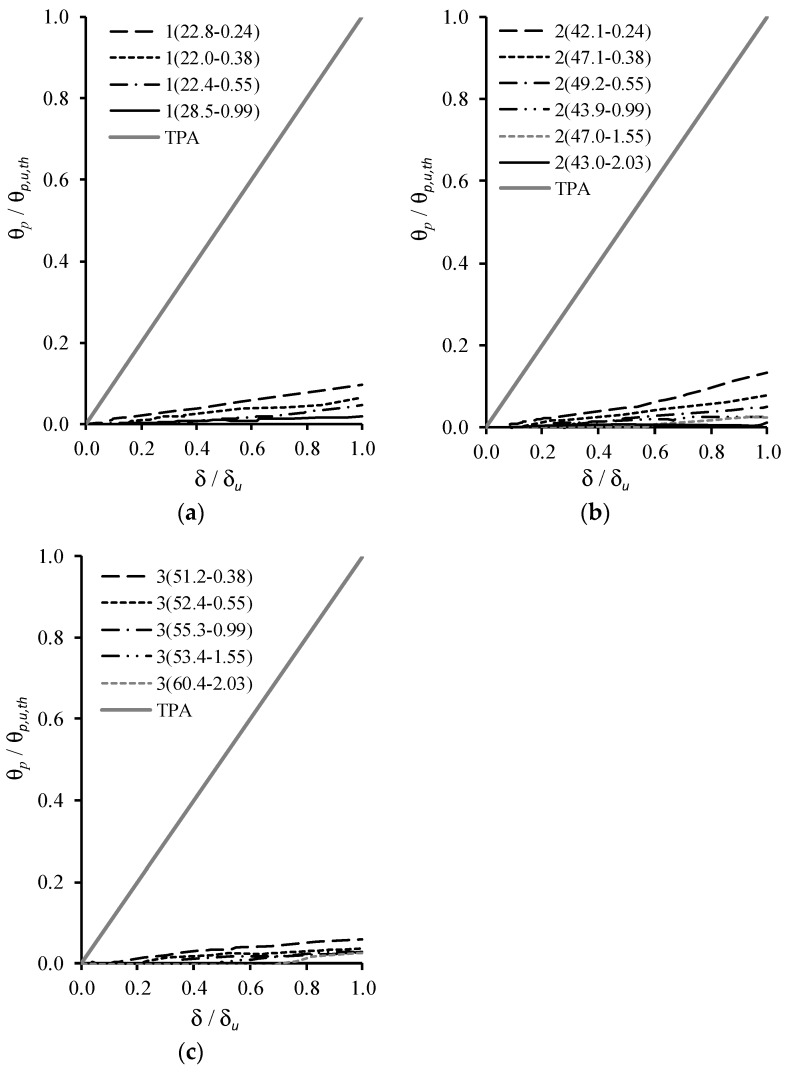
Plastic rotation versus deflection curves: (**a**) series 1; (**b**) series 2; (**c**) series 3.

Figure 5 represents, for each series of tested beams, the plastic rotation of the critical section (θ_*p*_) versus deflection at midspan (δ) curves. The non-dimensional axes are explained latter. Again, as explained before, the first beam of each series were not included because the last part of their P–δ curves were not considered reliable to obtain $\delta_{u}$. In addition, the longitudinal tensile reinforcement of Beam 3(51.6–2.69) yielded right before failure, so the failure was effectively brittle. For this reason, the behavior of this beam lies entirely in the elastic domain and was not included in Figure 5. A detailed explanation of how the graphs from Figure 5 were obtained can be found in [7]. However, a brief summary of the procedure used is given below.

As a first step, for each tested beam, the experimental and theoretical curves for the rotation of the critical section (θ) versus deflection at midspan (δ) were obtained. The experimental rotation was obtained by multiplying the experimental curvature in the critical section by a length equal to 1.2h, being h the height of the cross-section (Figure 1). According to Eurocode 2 [9], 1.2h corresponds to the length of the local plastic hinge of beams with ductile failure. The curvature was computed from the experimental strains measured along the height of the cross-sections in the failure zone, which were recorded from the external grid of Demec targets (Figure 2) by using a Demec strain gauge for several loading levels. From the experimental strains, and by assuming Bernoulli’s Hypothesis, an experimental average strain diagram was obtained through linear regression analysis. The angle of this diagram with the vertical is the curvature. The theoretical θ–δ curves were obtained from a theoretical elastic analysis (TEA), by using elasticity theory, and a theoretical plastic analysis (TPA) assuming a simple mechanism with a local plastic hinge at midspan.

As a second step, the elastic rotations computed from the TEA were subtracted to the experimental rotations θ to obtain the experimental plastic rotations $\theta_{p}$. In order to draw graphs with dimensionless axes, $\theta_{p}$ is divided by $\theta_{p,u,th}$ which represents the ultimate value of the theoretical plastic rotation computed from the TPA and corresponding to the ultimate experimental value of the deflection ($\delta_{u}$). In addition, δ is divided by $\delta_{u}$. From this procedure, the graphs represented in Figure 5 were drawn. Such graphs include the experimental curves and also the theoretical curves obtained from TPA. 

To characterize the plastic rotation capacity of the tested RC LWAC beams in the critical region, Bernardo et al. [7] defined an experimental parameter, called the plastic trend parameter (PTP). This parameter was obtained as follows.

From the graphs in Figure 5, two parameters called $C_{p,exp}$ and $C_{p,th}$ are computed. They represent, respectively, the areas below the experimental curve and below the theoretical curve (TPA). Parameter PTP is defined to be the ratio $C_{p,exp}/C_{p,th}$. This parameter estimates the experimental plastic rotation capacity level when compared with the theoretical one (TPA). The higher the value of PTP, larger the experimental plastic rotation capacity of the beam. Table 3 summarizes, for each tested beam, the values obtained for PTP.

## 4. Neutral Axis Depth versus Bending Moment at the Critical Sections

As referred before in the introduction section, for RC NWAC beams it is accepted that flexural ductility may be achieved through a correct design of the cross-section so that the relative neutral axis depth in failure (x/d) is small. This criterion is only valid for cross-sections under simple bending, such as the critical sections of the tested RC LWAC beams. In order to verify if this criterion is also valid for RC LWAC beams, the study of the evolution of the neutral axis depth through all loading history is important. In addition, the value of parameter x/d in failure and its variation with the ductility and the plastic rotation capacity is also important. The main objective of this section is to check if this evolution follows the same tendencies previously observed and accepted for RC NWAC beams. 

**Figure 6:**
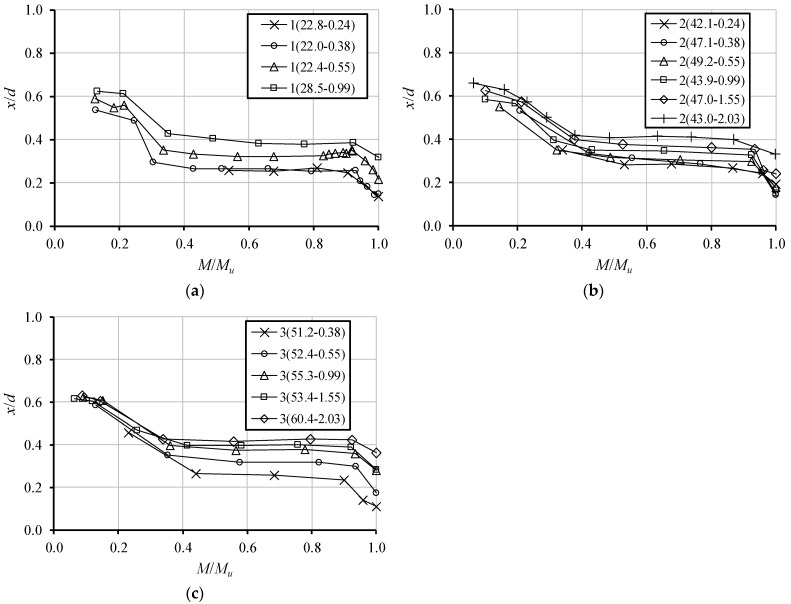
Neutral axis depth versus bending moment curves: (**a**) series 1; (**b**) series 2, (**c**) series 3.

Figure 6 represents, for each series of tested beams (same beams included in Figure 5), and for the critical section, experimental graphs with the evolution of the relative neutral axis depth (x/d) as function of the ratio $M/M_{u}$, being M the bending moment at the critical section of the tested beams and $M_{u}$ the ultimate bending moment (maximum moment). For a given beam and for each load level, the values of x/d at the critical section were calculated from the experimental average strain diagrams along the height of the section, which was obtained through a linear regression analysis of the measured strains with the external grid of Demec targets, as explained in Section 3.2.

From the graphs in Figure 6, three distinct zones for the evolution of the neutral axis depth can be observed as the bending moment increases.

In the first zone, x/d decreases as the bending moment increases. Since the critical region is under a positive bending moment, this observation is equivalent to stating that the neutral axis rises as the bending moment increases. In the uncracked stage, the neutral axis starts a little below to the mid height of the cross-section because of the influence of the longitudinal tensile reinforcement. This stage was hard to be recorded in the tested beams because the first crack in the critical section occurs for a low loading level, about 10 to 15% of the failure load. For most of the tested beams, the first measurement of the strains along the height of the cross-sections was performed after the appearance of the first crack. For this reason, x/d starts to decrease in the graphs in Figure 6. The first zone observed in the graphs corresponds to the development of a flexural crack which increases as the bending moment increases, both in depth and in width. 

In the second zone observed in the graphs from Figure 6, x/d tends to stabilize as the bending moment increases. This behavior is due to the cracking stabilization in the central region of the beams. At this stage, the main crack does not develop any further. Instead, new cracks are appearing as the bending moment increases, outside the critical section.

Finally, the third zone corresponds to a sudden decrease of x/d, or sudden rise of the neutral axis, up to the ultimate moment is reached in the critical section. This behavior starts when the longitudinal tensile reinforcement yields, which forces the main crack to develop even further due to the sudden rise of the strains in the steel bars.

The presented evolution of the neutral axis depth at the critical sections with loading agree with what was observed in previous studies with RC NWAC beams (for instance, Bernardo and Lopes in 2004 [10]).

Table 3 summarizes the values obtained for the relative neutral axis depth x/d. Two types of values are present, an experimental (${\left(x/d\right)}_{exp}$) and a theoretical (${\left(x/d\right)}_{th}$) one. The experimental value ${\left(x/d\right)}_{exp}$ was computed from the experimental average strain diagrams along the height of the critical section, assuming a conventional failure for the cross-section. It was assumed that the critical section reaches the conventional failure in bending when the maximum compressive strain in concrete reaches the ultimate conventional value stated from Eurocode 2 [9] for LWAC. This value is given by $\varepsilon_{lcu}=0.0035\eta_{1}$, where $\eta_{1}=0.4+0.6\delta_{l}/2200$ is a correction factor accounting for the dry density $\delta_{l}$ of LWAC (see Table 1). The theoretical value ${\left(x/d\right)}_{th}$ was computed from equilibrium and compatibility equations at ultimate limit state of the critical section. To define the compressive concrete stresses, the simplified method of rectangular stress block from Eurocode 2 [9] was used.

Table 3 shows that the theoretical values for the relative neutral axis depth, ${\left(x/d\right)}_{th}$, are remarkably smaller than the corresponding experimental ones, ${\left(x/d\right)}_{exp}$. This difference may be due to a certain inadequacy of the parameters which define the rectangular stress block in Eurocode 2 [9]. It may be possible that such parameters still need to be adjusted for LWAC, at least for the concrete type used in this study. It should be noted that using the lower values of ${\left(x/d\right)}_{th}$ for design, when compared to the experimental ones, will lead to lower values for the redistribution coefficient, as established in Eurocode 2 [9]. This will lead to an apparently higher redistribution capacity of the beams and shows the importance of the matter.

## 5. Analysis of the Neutral Axis Depth 

### 5.1. Evolution of the Neutral Axis Depth with Ductility and Plastic Rotation Capacity

**Figure 7:**
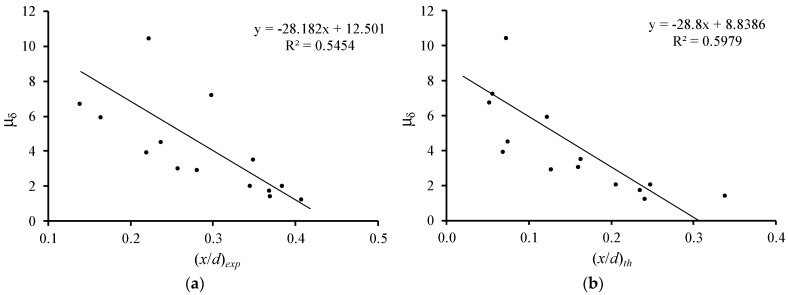
Neutral axis depth versus flexural ductility: (**a**) experimental; (**b**) theoretical.

**Figure 8:**
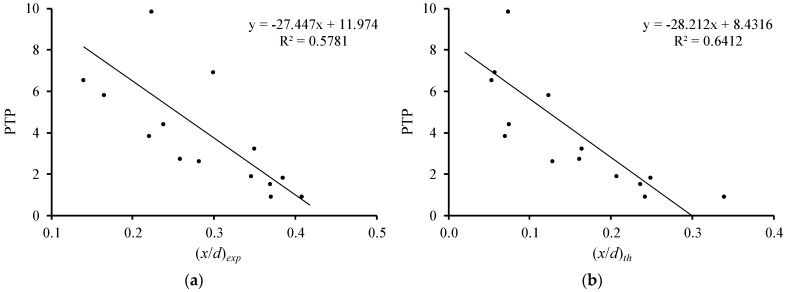
Neutral axis depth versus plastic rotation capacity: (**a**) experimental; (**b**) theoretical.

Figure 7 and Figure 8 illustrate the relations of the relative neutral axis depth (${\left(x/d\right)}_{exp}$ and ${\left(x/d\right)}_{th}$) with the flexural ductility ($\mu_{\delta }$) and with the plastic rotation capacity (PTP), respectively. In their previous studies, the authors observed that the influence of the compressive concrete strength in these parameters is small [6,7]. For this reason, all beams are included in the same graph, independently of their compressive concrete strength. Only beam 3(51.6–2.69) with no ductility was not included. The graphs in Figure 7 and Figure 8 include tendency lines to emphasize the evolution of the studied parameters.

Figure 7 and Figure 8 show that both ${\left(x/d\right)}_{exp}$ and ${\left(x/d\right)}_{th}$ parameters follow the same general tendency, which is that as the neutral axis depth at failure rises, both the flexural ductility and plastic rotation capacity decrease. This tendency was observed in previous studies and is accepted for RC NWAC beams (for instance [10]). Therefore, for the tested RC LWAC beams, the experimental results confirm the validity of the design rule used for RC NWAC beams to ensure flexural ductility and plastic behavior by limiting the neutral axis depth in the critical sections. 

### 5.2. Influence of the Longitudinal Tensile Reinforcement Ratio

**Figure 9:**
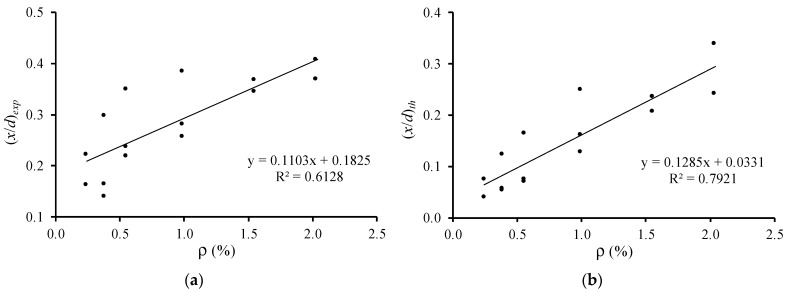
Neutral axis depth versus longitudinal tensile reinforcement ratio: (**a**) experimental; (**b**) theoretical.

Figure 9 illustrates the relations of the relative neutral axis depth (${\left(x/d\right)}_{exp}$ and ${\left(x/d\right)}_{th}$) with the longitudinal tensile reinforcement ratio (ρ). All beams are again included in the same graph, excepted Beam 3(51.6–2.69). Again, tendency lines are drawn to emphasize the evolution of the parameters studied.

Figure 9 shows that, for both the experimental and theoretical (x/d) parameters, the neutral axis depth at failure increases as the longitudinal reinforcement ratio increases. This observation explains why beams with higher longitudinal tensile reinforcement ratio presents lower ductility and plastic rotation capacity, as observed in Figure 4 and Figure 5. This confirms the previous observations from the authors [6,7]. Again, the observed tendency was also observed in other previous studies and is accepted for RC NWAC beams (for instance [10,11]. Therefore, for the tested RC LWAC beams, the experimental results confirm the validity of the additional design rule used for RC NWAC beams to ensure flexural ductility and plastic behavior, which limits the quantity of longitudinal tensile reinforcement in the critical sections. 

### 5.3. Influence of the Concrete Compressive Strength

To study the influence of the concrete compressive strength in the neutral axis depth, the tested beams must be grouped with similar longitudinal tensile reinforcement ratios. From Table 1, two groups with a relevant number of tested beams can be defined, namely:

- group A, with an average longitudinal reinforcement ($\rho_{m}$) equal to 0.47%, including Beams 1(22.0–0.38), 1(22.4–0.55), 2(47.1–0.38), 2(49.2–0.55), 3(51.2–0.38) and 3(52.4–0.55);
- group B, with an average longitudinal reinforcement ($\rho_{m}$) equal to 1.21%, including Beams 1(28.5–0.99), 2(43.9–0.99), 2(47.0–1.55), 3(55.3–0.99) and 3(53.4–1.55).

**Figure 10:**
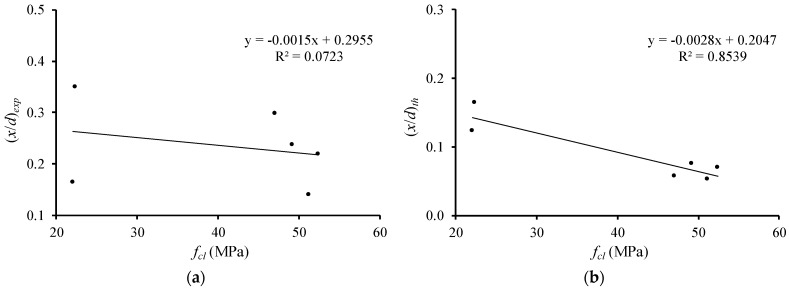
Neutral axis depth versus concrete compressive strength (group A): (**a**) experimental; (**b**) theoretical.

**Figure 11:**
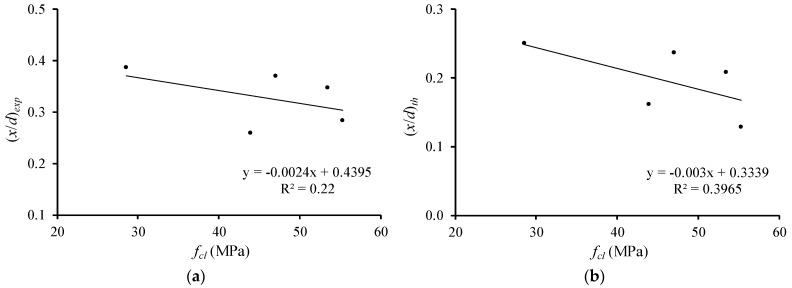
Neutral axis depth versus concrete compressive strength (group B): (**a**) experimental; (**b**) theoretical.

Figure 10 and Figure 11 illustrate the relations of the relative neutral axis depth (${\left(x/d\right)}_{exp}$ and ${\left(x/d\right)}_{th}$) with the concrete compressive strength for groups A and B, respectively. Again, tendency lines are drawn.

From Figure 10 and Figure 11, it can be seen that, for both the experimental and theoretical (x/d) parameters, the neutral axis depth at failure slightly decreases as the concrete compressive strength increases. This shows that the increase of the concrete compressive strength seems to be slightly favorable for the flexural ductility and plastic rotation capacity of the tested RC LWAC beams. When compared with the results of the previous section, it is clear that, among the two variable studies for the tested beams, the longitudinal reinforcement ratio is the most important. This also confirms the previous observations from the authors [6,7].

## 6. Conclusions 

In this article, an experimental study on the evolution of the neutral axis depth at failure in the critical cross-section of RC LWAC beams was presented. From the results obtained, the following main conclusions can be drawn:

- It was found that the evolution of the neutral axis depth for RC LWAC beams as the load increases is identical to the same reported and accepted for RC NWAC beams. This seems to indicate that the behavior of the formation and opening of the cracks for RC NWAC is also valid for RC LWAC;
- It was also found that both the flexural ductility and plastic rotation capacity of the RC LWAC beams increase as the neutral axis depth in failure decreases, as was also observed for RC NWAC beams. Therefore, the practical rule of limiting the neutral axis depth in the critical sections, to assure adequate flexural ductility and plastic rotation capacity levels, is also valid for RC LWAC beams;
- The results shown that the neutral axis depth in failure rises as the longitudinal tensile reinforcement ratio increases, as was observed and is accepted for RC NWAC beams. Therefore, the additional practical rule of limiting the quantity of longitudinal tensile reinforcement in the critical sections, to assure adequate flexural ductility and plastic rotation capacity levels, is also valid for RC LWAC beams;
- The results also show that the increase of concrete compressive strength seems to be favorable for both the flexural ductility and plastic rotation capacity for RC LWAC beams, since it was observed that the neutral axis depth at failure slightly decreases as the concrete compressive strength increases. However, the influence of the concrete compressive strength shown to be much lower than the longitudinal tensile reinforcement ratio;
- Finally, it was also found that the theoretical formulation from Eurocode 2 [9] based on the rectangular stress block diagram for concrete in compression, to compute the neutral axis depth at failure, does not give values approximate to those obtained from the experimental data. The theoretical values obtained were smaller when compared to those calculated experimentally. Therefore, the parameters to define the rectangular stress block as stated by Eurocode 2 [9] seems to still need to be adjusted for LWAC and some further research on this matter should be considered.

## Figures and Tables

**Table 1:** Properties of tested RC LWAC beams.

Beam	$f_{lc}$ MPa	$\delta_{l}$ kg/m^3^	$A_{s}$ cm^2^	d cm	ρ %
1(23.5–0.13)	23.5	1659	0.56 (2ϕ6)	27.7	0.13
1(22.8–0.24)	22.8	1685	1.01 (2ϕ8)	27.6	0.24
1(22.0–0.38)	22.0	1667	1.58 (2ϕ10)	27.5	0.38
1(22.4–0.55)	22.4	1651	2.26 (2ϕ12)	27.4	0.55
1(28.5–0.99)	28.5	1659	4.02 (2ϕ16)	27.2	0.99
2(45.1–0.13)	45.1	1802	0.56 (2ϕ6)	27.7	0.13
2(42.1–0.24)	42.1	1807	1.01 (2ϕ8)	27.6	0.24
2(47.1–0.38)	47.1	1809	1.58 (2ϕ10)	27.5	0.38
2(49.2–0.55)	49.2	1827	2.26 (2ϕ12)	27.4	0.55
2(43.9–0.99)	43.9	1788	4.02 (2ϕ16)	27.2	0.99
2(47.0–1.55)	47.0	1791	6.28 (2ϕ20)	27.0	1.55
2(43.0–2.03)	43.0	1790	8.04 (4ϕ16)	26.4	2.03
3(52.1–0.13)	52.1	1867	0.56 (2ϕ6)	27.7	0.13
3(51.2–0.38)	51.2	1879	1.58 (2ϕ10)	27.5	0.38
3(52.4–0.55)	52.4	1869	2.26 (2ϕ12)	27.4	0.55
3(55.3–0.99)	55.3	1910	4.02 (2ϕ16)	27.2	0.99
3(53.4–1.55)	53.4	1877	6.28 (2ϕ20)	27.0	1.55
3(60.4–2.03)	60.4	1953	8.04 (4ϕ16)	26.4	2.03
3(51.6–2.69)	51.6	1867	10.30 (2ϕ16 + 2ϕ20)	25.5	2.69

**Table 2:** Mix proportions of LWAC (contents per cubic meter).

Constituent Materials	Series 1	Series 2	Series 3
Portland cement 1 (kg): CEM II/B-L32.5N	335	-	-
Portland cement 2 (kg): CEM I 42.5R	-	445	494
Mineral addition 1 (kg): Limestone powder	-	-	35
Mineral addition 2 (kg): Microsilica	-	-	43
Superplasticizer (liters)	1.34	5.34	9.88
Water (liters)	174	146	153
Natural fine aggregate, Sand 0/5 (kg)	841	756	775
Lightweight coarse aggregate, Leca 4/12 (kg)	463	501	426
Water-binder ratio (in mass)	0.52	0.33	0.29

**Table 3:** Parameters to study the neutral axis depth.

Beam	$\mu_{\delta }$	PTP %	(*x*/*d*)_*exp*_	(*x*/*d*)_*th*_
1(22.8–0.24)	10.4	9.8	0.223	0.074
1(22.0–0.38)	5.9	5.8	0.165	0.123
1(22.4–0.55)	3.5	3.2	0.350	0.164
1(28.5–0.99)	2.0	1.8	0.385	0.249
2(42.1–0.24)	12.4	11.2	0.163	0.040
2(47.1–0.38)	7.2	6.9	0.299	0.057
2(49.2–0.55)	4.5	4.4	0.238	0.075
2(43.9–0.99)	3.0	2.7	0.258	0.161
2(47.0–1.55)	1.7	1.5	0.369	0.236
2(43.0–2.03)	1.4	0.9	0.370	0.339
3(51.2–0.38)	6.7	6.5	0.140	0.053
3(52.4–0.55)	3.9	3.8	0.220	0.070
3(55.3–0.99)	2.9	2.6	0.282	0.128
3(53.4–1.55)	2.0	1.9	0.346	0.207
3(60.4–2.03)	1.2	0.9	0.408	0.242
3(51.6–2.69)	≈1.0	≈0	0.585	0.374

## References

[1] Bogas J.A., Gomes A. (2013). Compressive behaviour and failure modes of structural lightweight aggregate concrete—Characterization and strength prediction. Mater. Des..

[2] Domagala L. (2011). Modification of properties of structural lightweight concrete with steel fibres. J. Civ. Eng. Manag..

[3] Cui H.Z., Lo T.Y., Memon S.A., Xu W. (2012). Effect of lightweight aggregate on the mechanical properties and brittleness of lightweight aggregate concrete. Constr. Build. Mater..

[4] Jung I.H., Yang W.J., Yi W.H., Jee S.W., Lee S.Y. Flexural Behavior of High-Strength Lightweight Concrete Beam with Eco Lightweight Aggregates. Proceedings of the 6th International Conference on Fracture and Damage Mechanics.

[5] Liu M.Y., Lin C., Li O., Ding Q.J., Hu S.G. Flexural Performance of Reinforced High-Strength Lightweight Concrete Beams. Proceedings of the 9th International Symposium on Structural Engineering for Young Experts.

[6] Bernardo L.F.A., Nepomuceno M.C.S., Pinto H.A.S. (2016). Flexural ductility of lightweight-aggregate concrete beams. J. Civ. Eng. Manag..

[7] Bernardo L.F.A., Nepomuceno M.C.S., Pinto H.A.S. (2016). Plastic rotation capacity of lightweight-aggregate concrete beams. J. Civ. Eng. Manag..

[8] Sin L.H., Huan W.T., Islam M.R., Mansur M.A. (2010). Reinforced lightweight concrete beams in flexure. ACI Struct. J..

[9] (2010). NP EN 1992-1-1. 2010. Eurocode 2: Design of Concrete Structures-Part 1: General Rules and Rules for Building.

[10] Bernardo L.F.A., Lopes S.M.R. (2004). Neutral axis depth versus flexural ductility in high strength concrete beams. J. Struct. Eng..

[11] Bernardo L.F.A., Lopes S.M.R. (2003). Flexural ductility of HSC beams. Struct. Concr..

